# Development and clinical implementation of a local automated kidney allocation platform integrating virtual crossmatch

**DOI:** 10.3389/fimmu.2026.1922544

**Published:** 2026-08-27

**Authors:** Mohammad Awaji, Saber AlZahrani, Rafah Bamrdouf, Dalal AlAbduladheem, Amani Mohammed, Mariam Alzahrani, Shaima Alkebasi, Bashaer Mohammad Alshallali, Abdullah Saad Alreaan, Sara Mohammed Bosbait, Sumayah Askandarani, Najib Ashoor Musaied, Khalid Beleed Akkari, Mohammed AlQahtani, Abdulnaser Mohammed Alabadi

**Affiliations:** 1 Histocompatibility and Immunogenetics Laboratory, King Fahad Specialist Hospital-Dammam, Dammam, Saudi Arabia; 2 Multiorgan Transplant Center of Excellence, King Fahad Specialist Hospital-Dammam, Dammam, Saudi Arabia; 3 Digital Health, King Fahad Specialist Hospital-Dammam, Dammam, Saudi Arabia

**Keywords:** cPRA, digital health, histocompatibility, HLA antibodies, kidney transplantation, organ allocation, sensitization, virtual crossmatch

## Abstract

**Background:**

Highly sensitized kidney transplant candidates possess preformed anti-human leukocyte antigen (HLA) antibodies that substantially reduce their likelihood of receiving a compatible deceased-donor organ. Manual virtual crossmatch (VXM) workflows are sequential and labor-intensive, often limiting histocompatibility laboratories to screening only a subset of eligible candidates during time-sensitive donor evaluations and potentially overlooking compatible recipients.

**Methods:**

We designed and developed an automated kidney allocation system (KAS) that links the hospital information system with HLA Fusion antibody data to perform candidate scoring and VXM prediction across the entire eligible active waitlist for every deceased donor workup. We conducted a single-center implementation study consisting of a cross-sectional analysis of 219 active kidney transplant candidates and a retrospective comparison of 67 deceased donor match runs processed by the automated KAS and the historical manual VXM workflow. The primary outcome was identification of potentially compatible highly sensitized patient (HSP) events. Analyse.

**Results:**

Of 219 candidates, 98 (44.7%) were highly sensitized (cPRA ≥80%). Elevated prevalence of HSP was associated with prior transplantation, pregnancy, and transfusion. HSP candidates waited far longer than non-sensitized peers (median 7.10 vs. 3.81 years; p < 0.001). Across 67 retrospective match runs, the automated KAS identified 13 potentially compatible HSP events compared with 4 under the manual workflow, more than threefold higher (rate ratio 3.25; 95% CI 1.06–9.97; p = 0.039). Automated runs completed in 17 ± 0.3 minutes, compared with 152 ± 1.6 minutes for manual workups (p<0.0001).

**Conclusions:**

A locally developed automated KAS substantially improved compatible donor identification for highly sensitized kidney transplant candidates while reducing VXM turnaround from hours to minutes. By enabling comprehensive evaluation of every eligible candidate during each deceased donor workup, automated allocation represents a practical and potentially scalable strategy that could improve equity and operational efficiency.

## Introduction

Kidney transplantation remains the ultimate treatment for end-stage renal disease, offering superior survival, better quality of life, and lower long-term healthcare costs than maintenance dialysis (1). Equitable access, however, is far from universal. Highly sensitized patients (HSP), those with preformed anti-human leukocyte antigen (HLA) antibodies through prior transplantation, pregnancy, or transfusion, remain among the most disadvantaged candidates on every waiting list. The breadth of these antibodies, estimated by the calculated panel reactive antibody (cPRA) score, directly predicts the probability that any given donor will be immunologically incompatible. Candidates with cPRA ≥80% are classified as highly sensitized; those with cPRA ≥98% face near-total exclusion from the deceased donor pool under conventional allocation (2–4).

Before targeted policy reforms, HSP candidates accumulated median wait times years longer than less-sensitized peers, with lower cumulative transplant probabilities and higher waitlist mortality (4–6). Recent advances in transplant informatics have enabled automated integration of clinical and immunologic data to support real-time allocation decisions. National allocation systems have demonstrated that systematic compatibility screening substantially improves transplantation opportunities for HSP, but such infrastructure is unavailable in many regions (7–13). Nonetheless, the final decision to accept or decline an organ still happens at the transplant center, often under acute time pressure and almost always limited by what the local histocompatibility laboratory can complete during a single offer window (14).

At the institutional level, the backbone for the decision is the virtual crossmatch (VXM): an in silico comparison of donor HLA antigens against the candidate’s antibody profile, derived from solid-phase single-antigen bead (SAB) testing reported as mean fluorescence intensity (MFI) (15, 16). When performed manually, VXM is inherently sequential. A technologist must pull each candidate’s antibody record and compare it to the donor antigen by antigen. In programs with a large sensitized fraction or with limited overnight staffing, only a portion of eligible candidates can realistically be screened per offer. The candidates who are not evaluated are not found incompatible; they are simply not assessed, which for a HSP recipient could mean missing a potentially rare compatible opportunity. Furthermore, SAB testing returns antibody specificities across hundreds of Class I and Class II HLA targets, and the clinical weight of any single donor-specific antibody (DSA) depends on its MFI and the broader immunologic context (17, 18). Translating that volume of data into reproducible VXM calls, candidate by candidate, by hand, is not realistic at scale.

This is the gap that institutional automation can fill. A system that integrates hospital information system (HIS) data with HLA antibody profiles in real-time can perform VXM prediction for the entire eligible waitlist within minutes, replacing a reactive, sequential workflow with a proactive decision-support process. The case for such tools is particularly strong in programs that do not sit within a high-volume national exchange, where deceased donor offers are scarce, and the cost of an unscreened compatible HSP candidate is high.

To address these challenges, we developed and implemented an institution-level automated KAS integrating HIS data with HLA antibody profiles to perform real-time candidate ranking and VXM prediction. This report had three objectives: to describe the system architecture and clinical implementation, to characterize sensitization patterns within our active kidney transplant waiting list, and to evaluate the impact of automated allocation on identification of potentially compatible highly sensitized candidates during deceased donor workups.

## Materials and methods

### Study design

This was a single-center study conducted at the King Fahad Specialist Hospital-Dammam, Saudi Arabia, to evaluate the locally developed automated KAS. The primary objective was to create a local system, at the center level, that improves the chance of HSP on the waiting list to find compatible donors. The project involved the development of the automated KAS platform for the local waiting list and conducting observational implementation to evaluate the system. The evaluation study consisted of a cross-sectional characterization of the active kidney transplant waiting list and a retrospective comparison of automated and historical manual deceased donor VXM workflows to assess the clinical and operational impact.

The primary outcome was the identification of potentially compatible HSP events during deceased donor match runs. Secondary outcomes included waiting list sensitization characteristics and match run duration.

The study comprises two datasets with different time anchors. The cross-sectional waitlist analysis characterizes the active waiting list as of December 2025 and was intended to describe the sensitization burden that motivated system development. The retrospective match run comparison uses all 67 deceased donor workups performed between January 2023 and December 2024, with automated match runs reconstructed using the candidate cohort and antibody profiles current at the time of each original workup rather than the December 2025 cohort.

### Automated kidney allocation system

#### System design and architecture

The automated KAS was designed as a single allocation interface that draws clinical, demographic, and immunologic data from multiple institutional information systems. The system retrieves recipient demographics, blood group, and viral serology from the HIS, while continuously synchronizing HLA antibody profiles, SAB specificities, and MFI values from the HLA Fusion software platform (One Lambda, Canoga Park, CA). HLA typing results, waitlist status, comorbidities, dialysis status, sensitization history, and cPRA scores are entered at recipient enrollment and updated as new immunologic data become available.

Data exchange between the HIS and KAS uses Health Level 7 messaging and customized application programming interfaces, implemented in line with institutional data governance standards and patient confidentiality regulations. A master patient index with standardized unique identifiers maintains record linkage across platforms. A dedicated validation layer flags missing or conflicting fields and routes them to the appropriate transplant coordinator or laboratory staff for review, reducing the risk of allocation decisions based on incomplete information.

**Figure 1:**
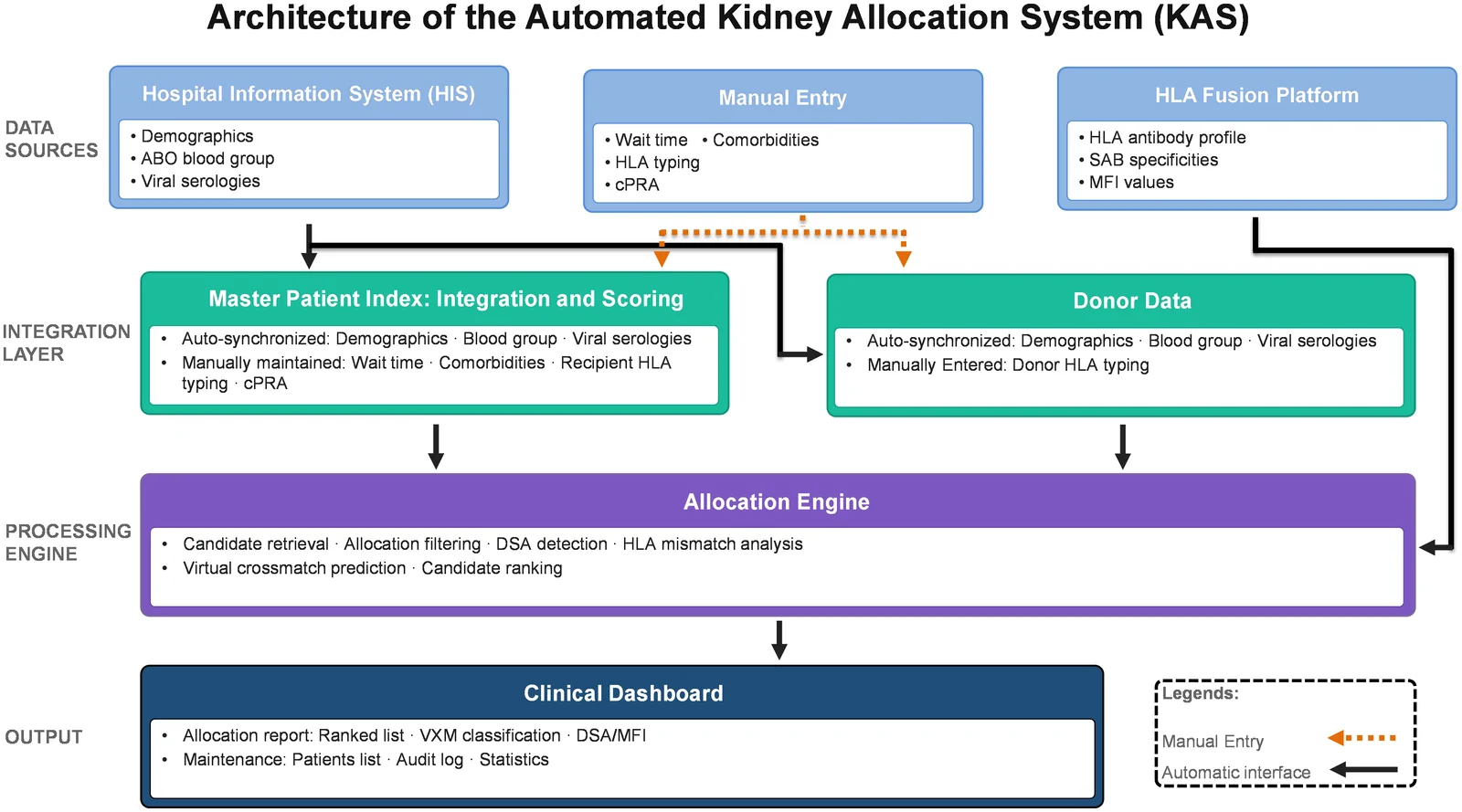
Architecture of the automated kidney allocation system (KAS). Recipient demographics, blood group, and viral serology data are automatically synchronized from the hospital information system (HIS) to a centralized master patient index through Health Level 7 based interfaces. Recipient waiting time, comorbidities, calculated panel reactive antibody (cPRA) scores and HLA typing are maintained manually within the master index, while HLA antibody profiles and mean fluorescence intensity (MFI) values are imported from HLA Fusion. Donor HLA typing is entered manually into the allocation engine, which performs automated candidate retrieval, donor-specific antibody (DSA) assessment, virtual crossmatch (VXM) prediction, and candidate ranking before presenting results through a clinical dashboard.

The user interface provides role-specific dashboards that display blood group compatibility, DSA status, VXM probability, and composite score for transplant physicians, coordinators, and laboratory personnel. MFI thresholds for VXM interpretation and the relative weighting of allocation criteria are configurable by authorized users, so institutional policy updates can be applied without software redevelopment (**Figure 1**).

#### Allocation algorithm

The allocation algorithm applies a sequential filter-and-score strategy modeled after principles used in contemporary allocation strategies while accommodating locally configurable criteria (8). Candidates are first grouped based on their blood types and can subsequently be filtered according to recipient age group (adult or pediatric) and transplant category (kidney only, kidney-pancreas, or kidney-liver). Each candidate is assigned a composite score incorporating cumulative wait time, recipient age group, and cPRA. Candidates are automatically ranked in descending order of this score. Additionally, candidates labeled with medical urgency for transplantation are brought to the top of the list by the system, regardless of their scores. **Table 1** shows the relative weights for the components of the composite score.

**Table 1:** Components of the composite score.

Composite score component	Relative wight
Wait time (Months)	0.1
Pediatric patient (Yes=1, No=0)	5
cPRA (0-50%) (Yes=1, No=0)	0.5
cPRA (51-70%) (Yes=1, No=0)	1
cPRA (71-80%) (Yes=1, No=0)	2
cPRA (81-90%) (Yes=1, No=0)	4
cPRA (91-95%) (Yes=1, No=0)	8
cPRA (96-98%) (Yes=1, No=0)	12
cPRA (>98%) (Yes=1, No=0)	16
Medical urgency (Yes=1, No=0)	Does not directly contribute to the score. When labelled as urgent, candidates automatically brought to the top of the list.

**Figure 2:**
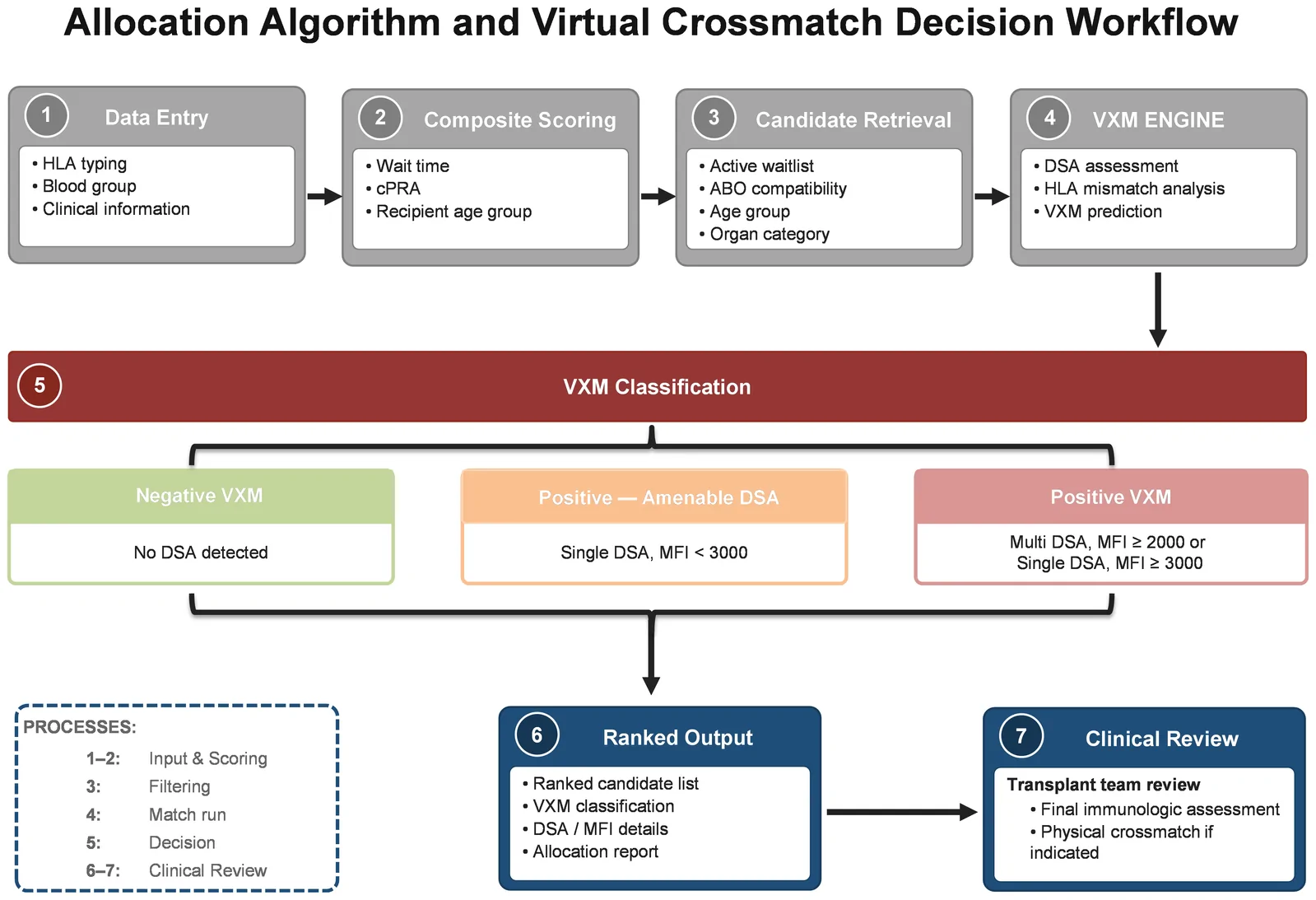
Allocation algorithm and virtual crossmatch decision. Following donor HLA entry, the system retrieves all active candidates, applies configurable allocation filters, performs automated donor-specific antibody (DSA) assessment, and classifies each candidate as negative virtual crossmatch (VXM), positive with amenable DSA, or positive VXM. Results are displayed as a ranked list of candidates for final clinical decision-making.

The VXM prediction module compares each candidate’s antibody profile against donor HLA typing using a three-tier classification. A negative VXM is assigned when no DSA is detected. A positive VXM is recorded when it detects one or more DSAs that exceed an MFI of 2,000. A positive with amenable DSA category, indicating a borderline result for individualized clinical judgement, is applied when a single DSA is present at MFI <3,000. This tiering allows rapid triage while preserving clinical discretion in equivocal cases, consistent with current guidance on MFI-based VXM interpretation (5, 13, 19). The module additionally calculates the number of HLA mismatches between the potential donor and recipients. The match-run output appears as a list of screened candidates ranked in descending order of their composite scores. The dashboard for each candidate displays demographics, viral reactivity, clinical urgency status, cPRA, HLA type and mismatch grade, and VXM prediction with DSA specificities with corresponding MFI values when present. The list can further be curated by VXM classifications: negative, positive, or positive with amenable DSA. Once the ranked, annotated list is generated, the allocation team reviews recipient-level detail to produce a short list for further final immunologic assessment. Unlike manual workflows, the automated algorithm evaluates every eligible candidate during every deceased donor workup irrespective of waiting list size (**Figure 2**).

#### System implementation and clinical workflow

The KAS was jointly developed by the Digital Health Department, Kidney Transplant Program, and Histocompatibility and Immunogenetics Laboratory. Development proceeded through staged integration testing using retrospective data, followed by validation in a dedicated sandbox environment before clinical deployment. This included validation of VXM predictions against experts’ evaluations for accuracy, reproducibility, and other quality assurance metrics; results of this validation were not included in this report. End-user training was performed for transplant coordinators, transplant physicians, and laboratory personnel. System modules are hosted on secure, institution-managed servers with role-based access controls aligned to each user’s operational scope. All allocation actions, manual overrides, and decision rationales are timestamped and logged, producing a complete audit trail for quality assurance and regulatory review.

During a deceased donor evaluation, the transplant coordinator enters donor demographics, blood group, and relevant medical history into the system. Histocompatibility laboratory personnel subsequently enter donor HLA typing, apply the relevant screening filters to gather the list of candidates, and execute the match-run. Within approximately 10–20 minutes, the system generates a complete score-ranked compatibility assessment for all eligible candidates together with predicted VXM classification and allocation score. The report can further be curated by VXM category: negative, positive, or positive with amenable DSA. The transplant team reviews candidates with negative or amenable VXM results and generates a shortlist of candidates to proceed for final immunologic assessment by the histocompatibility director or confirmatory physical crossmatching as clinically indicated based on the established internal policies and procedures (**Figure 2**). In addition to the score ranking and VXM results, other elements can contribute to selection of the shortlist include mismatch degree, geographical distance and readiness of the patient.

In contrast, with the legacy manual workflow, the clinical team pre-selects a list of candidates based on blood group compatibility, age group, and/or organ type that typically consists of approximately 15 patients; around 7 of them would be HSP. The list and donor HLA type are then sent to histocompatibility laboratory personnel for the subsequently VXM. The HLA technologist checks the donor’s HLA against the latest SAB results for each of the candidates and lists their DSAs with corresponding MFIs. The list is then sent to the histocompatibility director for VXM predictions and for assessing the need to physical crossmatches for each of the candidates. The clinical team then decides to proceed with candidates from the list or send a new list for another round.

### Waiting list management

Active kidney transplant candidates on the waiting list are typically maintained by quarterly SAB testing. A cutoff of 2,000 MFI is used in SAB results analysis where specificities with higher MFIs are considered positive. The cutoff can be adjusted in certain cases based on reactivity patterns such as reactivity to self-antigen or the presence of known spurious patterns. Positive specificities are listed for the major loci (A, B, C, DRB1, DQB1 and DPB1) at antigenic level unless a clear allele-specific pattern is recognized. Specificities to DQA1 and DPA1 are only listed when their corresponding paired DQB1 or DPB1 alleles are excluded, and they are listed at the allelic level. The final assignment following analysis are therefore considered an unacceptable antigen list to be used for VXM prediction and for cPRA calculation through (https://optn.transplant.hrsa.gov/data/allocation-calculators/cpracalculator). The automated systems utilized the most recent sample for the VXM analysis; historic results are also considered in the final assessment by the histocompatibility personnel.

### Waitlist analysis

All active kidney transplant candidates listed as of December 2025 were included. Candidates temporarily inactive or removed from the waiting list before the study date were excluded. A total of 219 candidates met these criteria. Variables of interest included age (adult ≥18 years vs. pediatric <18 years), sex, blood group, prior transplantation, pregnancy history (among female candidates), and transfusion history. cPRA was taken from the most recent antibody assessment on file. Highly sensitized status was defined as cPRA ≥80%. This analysis aimed to provide a snapshot of the HSP distribution in our waiting list.

### Retrospective match run comparison

To evaluate the clinical and operational impact of the automated KAS, retrospective automated match runs were performed for deceased donor evaluations previously processed using the manual workflow. In the automated match-runs, we included all eligible candidates at the time of the original match-run, typically blood group-matched patients. The study included all 67 deceased donor workups performed between January 2023 and December 2024. A potentially compatible HSP event was defined as the identification of any HSP candidate with a negative or amenable VXM result. Events were counted for both workflows, event rates per run were calculated, and the incidence rate ratio (IRR) comparing automated to manual workflow was estimated by Poisson regression with 95% confidence intervals. Moreover, a paired donor match run analysis was conducted to compare the number of match runs that produced potentially compatible HSP events using the McNemar test. Number of total candidates, number of HSP candidates per match run, and durations of match runs were compared using the Wilcoxon test. Match run duration was recorded prospectively for the automated workflow and reconstructed from contemporaneous laboratory records for the manual workflow and is defined as the time between when the on-call technologist starts performing VXM and when the report is submitted to the clinical coordinator/physician.

### Statistical analysis

Associations between categorical variables and HSP status were evaluated with Fisher’s exact test. cPRA distributions and waiting times between groups were compared using the Mann–Whitney U test. The effect of automation on potentially compatible HSP identification was estimated using Poisson regression with a log link, and results were reported as IRR with corresponding 95% confidence intervals (CI). Paired donor match run analyses were compared using the Wilcoxon test for the number of candidates and the match run durations, and using the McNemar test for the number of match runs that produced potentially compatible HSP events.

Statistical analyses were performed using GraphPad Prism 10.4.1. Two-sided tests were used throughout, and statistical significance was defined as p < 0.05.

## Results

### Waitlist characteristics and sensitization distribution

Of 219 candidates on the active waiting list, 98 (44.7%) met criteria for highly sensitized status (cPRA ≥80%). Demographic and clinical correlates are summarized in **Table 2**.

**Table 2:** HSP distribution in the waiting list.

Characteristic	Group	%HSP (n) within group	P-value
Age group	Adult (n = 196)	49.0%(96)	0.0002
	Pediatric (n = 23)	8.7%(2)	
Gender	Female (n = 123)	56.1%(69)	0.0002
	Male (n = 96)	30.2%(29)	
History of Transplant	Yes (n = 52)	92.3%(48)	<0.0001
	No (n = 167)	29.9%(50)	
History of Pregnancy*	Yes (n = 83)	71.1%(59)	0.00023
	No (n = 31)	32.3%(10)	
History of Transfusion	Yes (n = 33)	60.6%(20)	0.025
	No (n = 135)	37.8%(51)	
	Unknown (n = 51)	52.9%(27)	
Blood group	A (n = 45)	48.9%(22)	0.587
	AB (n = 20)	50.0%(10)	
	B (n = 51)	49.0%(25)	
	O (n = 103)	39.8%(41)	

*Pregnancy history analyzed among female candidates only (n = 114). HSP, highly sensitized patient.

Adults were more often sensitized than pediatric candidates (HSP: 49.0% vs. 8.7%; p = 0.0002), in keeping with the greater cumulative alloimmune exposure of older patients with more complex histories. Sensitization was also more frequent in women than in men (HSP: 56.1% vs. 30.2%; p = 0.0002), reflecting, in large part, pregnancy-related antibody formation.

**Figure 3:**
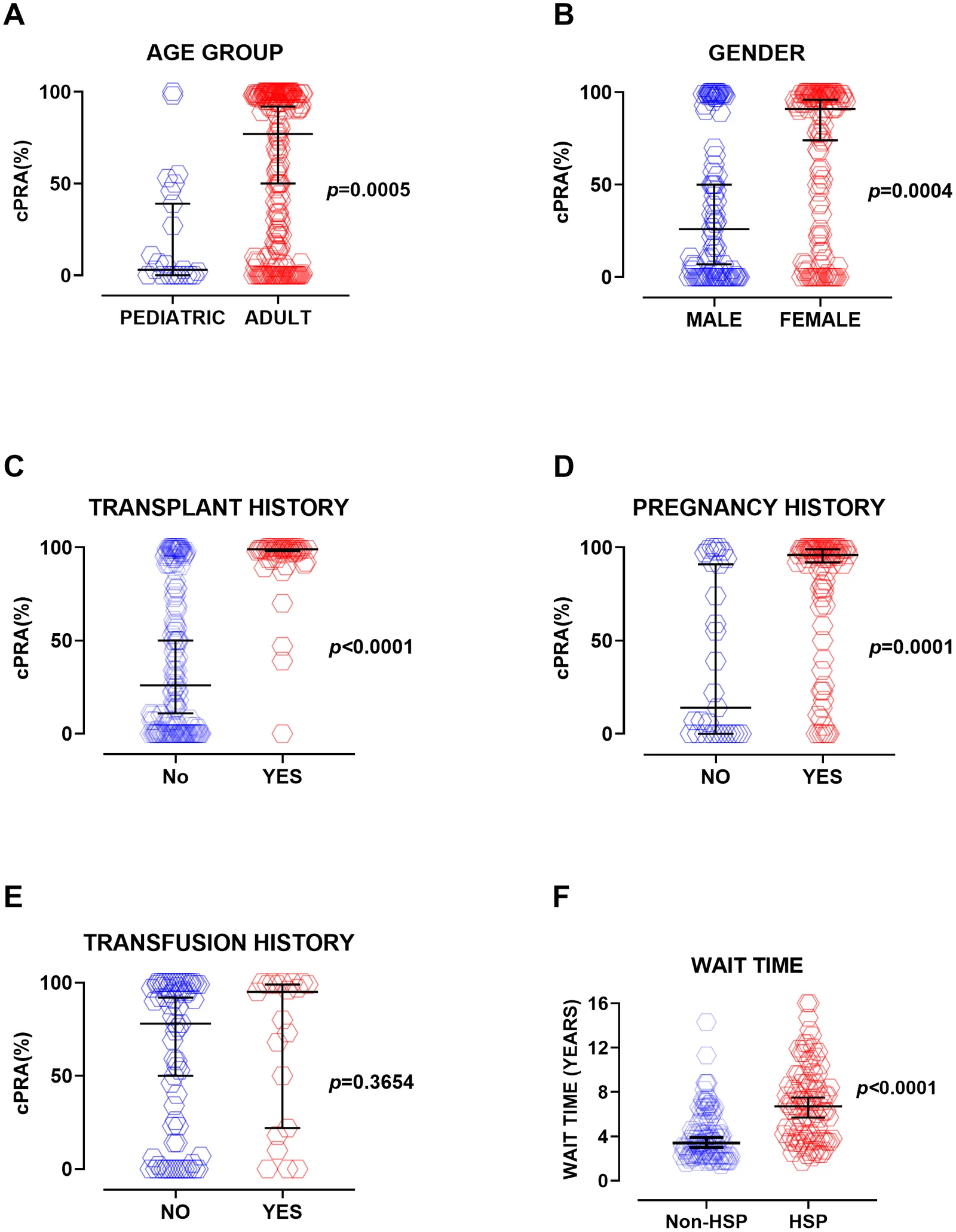
Association of recipient characteristics with sensitization status and waiting time for kidney transplantation. Distribution of calculated panel reactive antibody (cPRA) according to **(A)** age group (pediatric vs. adult), **(B)** sex, **(C)** previous kidney transplantation, **(D)** pregnancy history among female candidates, and **(E)** transfusion history. **(F)** Comparison of waiting time between highly sensitized patients (HSP; cPRA ≥80%) and non-HSP candidates. Each point represents an individual candidate; horizontal lines and error bars indicate median and interquartile range. Statistical significance was assessed using the Mann–Whitney U test.

Prior transplantation was the strongest single correlate of HSP status: 92.3% of candidates with a prior transplant were highly sensitized, compared with 29.9% of those without (p < 0.0001). Among women, history of pregnancy raised sensitization from 32.3% to 71.1% (p = 0.00023). Transfusion history was also associated with HSP status, though less strongly (60.6% vs. 37.8%; p = 0.025). Blood group distribution had no significant effect (p = 0.587) (**Table 2**). Comparing the median cPRA across groups, we arrived at the same conclusion, except for transfusion history, which showed no statistically significant difference (**Figure 3**).

The wait-time gap was significant: HSP waited a median of 7.10 years for a kidney transplant, compared with 3.81 years for non-highly sensitized candidates (p < 0.001), confirming that the access disparity well documented in larger registries persists within our own program (**Figure 3**).

### Impact of the automated KAS on potentially compatible HSP identification

Before implementation, deceased donor workups relied on manual VXM, with a technologist reviewing each candidate’s antibody profile against the donor HLA type one at a time. The process typically ran 2–3 hours per workup (median = 152 minutes), often into overtime, and in practice could only cover a fraction of the waitlist during any single offer (median of 14 candidates per run and including a median of 7 HSP candidates).

To assess if the automated KAS would improve identification of potentially compatible HSP events. Applying the automated KAS retrospectively to 67 deceased donor evaluations, previously performed manually, yielded a clear improvement in potentially compatible HSP identification. The manual workflow had identified 4 (2 negative and 2 amenable) potentially compatible HSP events in 2 runs across 67 runs (0.060 events per run). The automated KAS identified 13 events (6 negative and 7 amenable) in 9 runs across the same donors (0.194 events per run). Poisson regression gave a IRR of 3.25 (95% CI 1.06–9.97; p = 0.039) in favor of the automated workflow. Paired analysis demonstrated that the automated workflow was able to identify potentially compatible events in 9 runs (7 out of 9 were unique, identified only by the KAS) whereas the manual workflow was not able to identify any unique events (p=0.023); **Table 3**. All additional events identified with automated workflow belong to candidates who were not assessed by the manual workflow.

**Table 3:** Comparison between manual and automated reviews.

Measure	Manual review	Automated review	p value
Match runs with potentially compatible HSP events. (n=67 match run)	2	9	p=0.023
Total potentially compatible HSP events. Events(Amenable events)	4(2)	13(7)	p = 0.039
Number of candidates per match run Median (IQR)	14 (IQR 14-16)	45 (IQR 38-104)	p<0.0001
Number of HSP candidates per match run Median (IQR)	7 (IQR 6-8)	22 (IQR 20-41)	p<0.0001
Match run duration (minutes) Mean ± SE	152 ± 1.6	17 ± 0.3	p<0.0001

Automated runs are typically completed in 10–20 minutes (mean in minutes ± SE: 17 ± 0.3), against 2–3 hours (mean in minutes ± SE: 152 ± 1.6) for the corresponding manual workups (p<0.0001); **Table 3**.

## Discussion

In this report, we describe the development of a locally built automated KAS that more than tripled the rate of potentially compatible donor identification for HSP and cut histocompatibility laboratory turnaround from hours to minutes. These findings add to a growing body of evidence that institution-level digital tools can meaningfully complement, or mirror, national policy in addressing the persistent access disadvantage faced by sensitized candidates.

The sensitization profile of our waitlist is consistent with what one would expect of a transplant population shaped by prior grafts, pregnancies, and transfusions (20–23). The wait time gap of 7.10 versus 3.81 years between HSP and non-HSP candidates mirrors pre-reform reports from larger North American and European registries (9, 13). That such a gap persists within a single program with active local oversight underscores how immunologic barriers translate into measurable inequity regardless of the allocation tier at which a program operates.

The mechanism by which the automated KAS produced its rate ratio of 3.25 is not subtle: by performing VXM prediction across the entire eligible waitlist within minutes, the system removes the practical ceiling that manual review imposes on candidate coverage. Under manual conditions, time and staffing constraints inevitably mean that many of the potentially compatible HSP candidates are not screened at all during a given offer. This effect is most pronounced in programs with large sensitized fractions and constrained overnight staffing. Moreover, in our setting in Saudi Arabia, the scarce deceased donor offers and the absence of a centralized national allocation platform shift the responsibility of mitigating HSPs chances to the individual transplant centers (24).

The U.S. KAS reform of 2014 produced a 34.5% increase in deceased donor transplant rates for candidates with cPRA 98–100% in its first year (8, 13). The Eurotransplant Acceptable Mismatch program has driven transplant rates for sensitized patients to levels approaching those of non-sensitized candidates by performing proactive, registry-wide HLA compatibility screening (9). The Scandiatransplant program reported more than a doubling of transplant probability for HSP after systematic implementation (11). We cite these programs for the underlying principle that comprehensive simultaneous screening maximizes the probability of identifying a compatible match, and not as a comparator. These programs operate at a scale and degree of centralization that no single institution can replicate. But they share a unifying principle that systematic, simultaneous immunologic screening of all eligible candidates maximizes the probability of identifying compatible matches. The local KAS described here applies that principle precisely.

In our region, where overall deceased donor volumes are modest, each donor workup with missed compatible HSP events carries disproportionate weight. It accumulates HSP further on the waiting list, increasing their likelihood for additional sensitization events. An institutional system that ensures every active, eligible candidate is screened against every offer is, in this context, the lever for potentially improving HSP access.

The operational gains are themselves clinically consequential. Reducing match run duration from hours to minutes, if we would speculate, could have an impact on cold ischemia time, organ utilization, and the sustainability of the laboratory workforce. As transplant programs face increasing demand for rapid immunologic assessment with limited staffing, the ability to execute a comprehensive, reproducible VXM across an entire waitlist in under twenty minutes is a real efficiency gain. Although the following variables were not assessed statistically, we could suggest that the automation may potentially also reduce inter-technologist variability, lower clerical error rates, and free experienced staff to focus on interpretive and complex-case work that genuinely requires human judgment. From an economic standpoint, reduced overtime during urgent evaluations can translate to direct labor savings, and improved HSP identification has the potential to offset the higher costs of prolonged dialysis and repeated transplant evaluations for candidates who remain on the list (1, 25, 26).

Several limitations deserve acknowledgment. This is a single-center, retrospective analysis over a limited number of donor evaluations; transplant outcomes were not evaluated as part of the present report. The VXM algorithm relies on MFI-based thresholds that, while consistent with widely accepted practice and published guidelines, inherit the known limitations of SAB-based antibody assessment: MFI values vary technically between runs, and MFI level does not always correlate with complement activation or with a positive physical crossmatch (17, 18, 27, 28). C1q and C3d binding data are not currently integrated, nor is eplet-level analysis (29, 30). The system’s modular architecture is designed to benefit by mitigating the local sensitization burden, baseline workflow efficiency, and the degree to which the manual workflow was previously rate-limited. Programs with already-automated processes or different waitlist compositions may see smaller or differently distributed gains. Lastly, multicenter validation will be essential before generalizable benefits can be claimed.

Future development and enhancement will work along several fronts. Integration of contemporary matching tools HLAMatchmaker and PIRCHE could refine compatibility assessment beyond the antigen-level resolution of MFI thresholds, potentially opening the donor pool to candidates with complex antibody profiles currently excluded on antigen-level DSA (31, 32). Allele-level HLA typing will refine matching further as it becomes routinely available. Longitudinal linkage of allocation decisions to graft and patient outcomes will enable evidence-based recalibration of MFI thresholds and allocation weights. In the longer term, machine learning models trained on local donor–recipient–outcome data may yield individualized VXM risk estimates that move beyond static thresholds, particularly for borderline DSA scenarios.

## Conclusion

Institutional automation of the VXM workflow improved the identification of potentially compatible donors for HSP. By making it feasible to evaluate every eligible candidate during every deceased donor offer, the system removes a structural ceiling on HSP access to match runs that no amount of clinical effort can overcome by hand. Locally developed, institution-level automated allocation tools represent a pragmatic solution. Its scalability and ability to improve equity and outcomes requires prospective multicenter evaluation.

## Data Availability

The raw data supporting the conclusions of this article will be made available by the authors, without undue reservation.
